# The Manifesto of the Voluntary System

**Published:** 1921-02-05

**Authors:** 


### The Hospital

The Workers' Newspaper of Administrative Medicine and Institutional Life, Administration, National Insurance and Health.

No. 1809, Vol. LXIX. SATURDAY, FEBRUARY 5, 1921. PRICE TWOPENCE

### THE MANIFESTO OF THE VOLUNTARY SYSTEM.

By far and away the most important document dealing with the position of the voluntary hospitals of this country that has seen the light for many months is the report of the Executive Committee of King Edward's Hospital Fund for London, which led to the thirteen resolutions passed by the General Council of the Fund, to be found on another page of this week's issue of The Hospital.

These resolutions are a crystalised form of the no mean accumulation of knowledge of hospital affairs and conditions at the disposal of a great hospital central body, strengthened by financial data describing the position of the hospitals of London up to the close of 1920. No other body is able, let alone qualified, to speak on the subject with the authority and precision which characterise an important and epoch-making document.

It is peculiarly grateful to those entrusted with the conduct of this journal, as it would have been to its founder and late editor, to see that all he strove for and all we are striving to attain—namely, the preservation of the great voluntary system of hospitals in this country—is in the forefront of the manifesto of the King's Fund. It is none the less grateful to remark that the policy proposed to bring about this object is practically a summary of the policy urged in these columns from the very commencement of what is called "the hospital crisis." And with the gentlest qualification in one or two particulars we are able to accept the principles of policy to be recommended to His Majesty's Government by King Edward's Fund as identical with those we are willing to continue to struggle for to the full extent of our ability.

What we predict will be known for a long time in the hospital world as "the thirteen resolutions" are marked by an absence of that obliquity of diction which is the province of the politician. In clear and almost homely English we are told that the voluntary method provides at the least cost the best medical and surgical treatment combined with advance in medical knowledge and practice, and are advised not only what to consider as the best means of raising funds to maintain the system, but as to broad methods of expending these funds to the greatest advantages. Subject to a preponderant voluntary element in the income, certain forms of Government help or co-operation are suggested. Amongst these is "some form of assistance based on the amount received for the benefit of hospitals from voluntary sources," or, in other words, something of the Australian pound-for-pound method of State help, which has been of doubtful success, and should be more closely inquired into before it is either advocated or adopted. Payment by Government or other public authorities in respect of the treatment of any classes of patients for whom those authorities have taken responsibility has always had the strongest advocacy in these pages, and requires no further endorsement at the present moment. That some of these payments should be dated back for a period, in view of the large accumulations of National Health Insurance funds, is a matter of common justice, considering that these funds have largely been built up by means of hospital service.

Central organisation for Metropolitan and provincial hospitals is proposed, and quite properly the organisation of the King's Fund is mentioned as the possible basis of a central body for the Metropolitan area. The General Council of King Edward's Fund is a body which for the purpose, did we seek a ready-made Central Council, could not, for prestige or for knowledge of hospital affairs, be equalled. It already has disciplinary power by means of the substantial funds placed in its discretion to administer. But it is self-electing—a feature in a democratic age which would not make it acceptable to the majority of hospitals were it endowed with increased powers and given a controlling voice in the allocation of State or local-government moneys. But this probably is an aspect of the matter which has not escaped the analytic observation of the Fund's advisers.

We have thought well to select from the report of the Executive Committee (see p. 439) the enlightening passages that deal with the financial position of the hospitals of London at the end of 1920. It will be seen that in the general picture it is shown that 113 hospitals collectively exceeded their income last year by £394,000—that is, without counting the quarter of a million emergency grant provided out of accumulated funds by King Edward's Fund. But forty-two hospitals actually had a balance in hand (presumably apart from the emergency grant) as a result of the year's working. If the gross result is as we suspect—that the hospital financial position is greatly overshadowed by the startling deficiencies of some of the larger general hospitals with medical schools—namely, the hospitals that notoriously cost more to maintain than the others—the result is that unless extreme care is taken to examine the true position, the main body of voluntary hospitals might be hurried into a changed system which they do not desire, and which they have not contributed to bring about.

